# The natural compound Guttiferone F sensitizes prostate cancer to starvation induced apoptosis via calcium and JNK elevation

**DOI:** 10.1186/s12885-015-1292-z

**Published:** 2015-04-11

**Authors:** Xin Li, Yuanzhi Lao, Hong Zhang, Xiaoyu Wang, Hongsheng Tan, Zhixiu Lin, Hongxi Xu

**Affiliations:** 1 School of Pharmacy, Shanghai University of Traditional Chinese Medicine, Shanghai, 201203 P.R. China; 2 Engineering Research Center of Shanghai Colleges for TCM New Drug Discovery, Shanghai, 201203 P.R. China; 3 School of Chinese Medicine, Faculty of Science, The Chinese University of Hong Kong, Shatin, N.T, Hong Kong, SAR China

**Keywords:** Guttiferone F, Prostate cancer, Apoptosis, Starvation, Natural compound

## Abstract

**Background:**

In a cytotoxicity screen in serum-free medium, Guttiferone F showed strong growth inhibitory effect against prostate cancer cells.

**Methods:**

Prostate cancer cells LNCaP and PC3 were treated with Guttiferone F in serum depleted medium. Sub-G1 phase distributions were estimated with flow cytometry. Mitochondrial disruption was observed under confocal microscope using Mitotracker Red staining. Gene and protein expression changes were detected by real-time PCR and Western blotting. Ca^2+^ elevation was examined by Fluo-4 staining under fluorescence microscope. PC3 xenografts in mice were examined by immunohistochemical analysis.

**Results:**

Guttiferone F had strong growth inhibitory effect against prostate cancer cell lines under serum starvation. It induced a significant increase in sub-G1 fraction and DNA fragmentation. In serum-free medium, Guttiferone F triggered mitochondria dependent apoptosis by regulating Bcl-2 family proteins. In addition, Guttiferone F attenuated the androgen receptor expression and phosphorylation of ERK1/2, while activating the phosphorylation of JNK and Ca^2+^ flux. Combination of caloric restriction with Guttiferone F *in vivo* could increase the antitumor effect without causing toxicity.

**Conclusions:**

Guttiferone F induced prostate cancer cell apoptosis under serum starvation *via* Ca^2+^ elevation and JNK activation. Combined with caloric restriction, Guttiferone F exerted significant growth inhibition of PC3 cells xenograft *in vivo.* Guttiferone F is therefore a potential anti-cancer compound.

**Electronic supplementary material:**

The online version of this article (doi:10.1186/s12885-015-1292-z) contains supplementary material, which is available to authorized users.

## Background

Prostate cancer (PCa) is the most commonly diagnosed cancer in men and one of the leading causes of cancer death in the United States [1,2]. For patients with localized prostate cancer, radical prostatectomy, chemotherapy and radiation therapy result in prolonged survival. In early stages, prostate cancer proliferation increases with androgens stimulation. Patients at this stage can be treated with androgen ablation therapy by decreasing circulating androgens or by blocking the androgen receptor using antiandrogens [3,4]. However, the majority of patients eventually progress to a state of castration-resistant prostate cancer (CRPC). The current treatment modality for those patients with CRPC is chemotherapy based on docetaxel, which provides minor improvements in survival rate [5].

Diet and obesity are important factors contributing to prostate cancer development. Recent reports showed that dietary patterns and food constituents could affect cellular activity and gene expression [6-8]. Specifically, western-style diets enriched in fat and cholesterol could accelerate PCa progression [9,10]. In fact, nutrient plays an important role in cancer cell survival and progression [11]. Therefore, screening active compounds in nutrient deprived cells may be an alternative way for anticancer drug development. For instance, Awale *et al*. reported that using the nutrition depleted medium screen platform, natural compound arctigenin could eliminate the tolerance of pancreatic cancer cells to nutrient starvation. Targeting nutrition deprived PCa may be a novel strategy in anticancer drug development.

*Garcinia* species (Family *Guttiferae*) are tropical evergreen trees and shrubs distributed in Southeastern Asia [12]. Xanthones and benzophenone derivatives are the major bioactive components [13-16]. The *Garcinia* resin, gamboge, has been used by Chinese medicine practitioners to treat inflammation and promote detoxification [14,17]. In addition, the compounds isolated from many *Garcinia* species showed various bioactivities, such as antitumor, anti-inflammatory, antibacterial, antioxidant, antiviral and neuroprotective effects [12,15,18-22].

**Figure 1:**
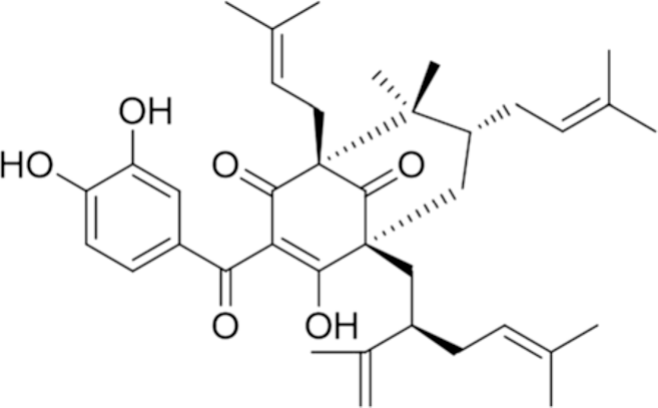
The structure of guttiferone F (C_38_H_50_O_6_, molecular weight: 602.8).

Guttiferone F (GF) is a prenylated benzophenone derivative (Figure 1) firstly isolated from *Allanblackia stuhlmannii* [23], Recently, we reported that GF, isolated from the twigs of *Garcinia esculenta,* could induce caspase-3 mediated apoptosis in HeLa cells [24]. In this study, we found that GF could significantly activate mitochondria dependent apoptotic signal under nutrient deprivation, but not affecting the cells in normal culture medium. Interestingly, *in vivo* study showed that caloric restriction could enhance the antitumor effect of GF in PCa xenograft model.

## Methods

### Cell culture

LNCaP, PC3, HepG2, HeLa and CNE cells were obtained from ATCC (Rockville, MD, USA). LNCaP and PC3 cells were maintained in RPMI1640 (Sigma-Aldrich) supplemented with 10% fetal bovine serum (FBS) (Invitrogen, St. Louis, MO, USA). HepG2, HeLa and CNE Cells were maintained in DMEM (Sigma-Aldrich) supplemented with 10% FBS. The cells were maintained in a humidified atmosphere containing 5% CO_2_ at 37°C. For nutrient starvation, the medium with serum was removed and washed by PBS for three times and then serum free RPMI1640 was applied.

### Cell viability assay

The cell viability was assessed by MTT assay [25]. Cells were seeded in 96-well plates and treated with Guttiferone F at different concentrations. Cell viability was measured 48 h after drug treatment. Cells were incubated with 100 μl of fresh medium containing 10 μl of 3-(4,5-Dimethylthiazol-2-yl)-2,5- diphenyltetrazolium bromide (MTT, Sigma, St. Louis, MO, USA) and subsequent dissolving of formazan crystals in DMSO. Absorbance was measured at 570 nm by microplate reader. The absorbance of untreated cells in medium was considered as 100% survival.

### Flow cytometry

Cells were fixed in 70% ethanol in PBS overnight. For cell cycle distribution, cells were counterstained with propidium iodide (Sigma) and analyzed for their DNA content using BD FACSCalibur flow cytometry as described previously [26].

### Live-cell imaging

For mitochondrial staining, LNCaP cells grown on coverslips were stained with 50 nM MitoTracker Red (Invitrogen) in pre-warmed medium for 30 min at 37°C. All of the samples were examined under a FluoView FV10i confocal microscope (Olympus Corporation, Tokyo, Japan).

### Western blotting

Western blotting analysis was carried out as previously described [25]. Cells were lysed in ice-cold whole cell extract buffer (50 mM pH8.0 Tris–HCl, 4 M urea and 1% TritonX-100), supplemented with complete protease inhibitor mixture. Cell extracts were resolved by SDS-PAGE gel electrophoresis and transferred to a polyvinylidene fluoride membrane. After blocking with 5% non-fat milk in Tris-buffered saline containing 0.2% Tween-20, the membranes were probed with the following antibodies: PARP (Cell signaling, #9542), total and cleaved caspase-3 (Asp175) (Cell signaling, #9662/#9664), total and cleaved caspase-9 (Asp330) (Cell signaling, #9502/#7237), caspase-7 (Cell signaling, #9492), Bax (Cell signaling, #5023), Bcl-xL (Cell signaling, #2764), Bcl-2 (BD Biosciences, #551107), Phospho-ERK (Thr202/Tyr204) (Cell signaling, #4370), total ERK (Cell signaling, #4695), Phospho-JNK (Thr183/Tyr185) (Cell signaling, #4668), total JNK (Cell signaling, #9252), AR (Cell signaling, #5153) and β-actin (Cell signaling, #2118). Following incubation with horseradish peroxidase coupled secondary anti-mouse (KPL, Gaithersburg, MD, USA) or anti-rabbit antibodies (KPL), protein bands were visualized using an enhanced chemiluminescence kit (Pierce, Rockford, IL, USA). β-actin was used to ensure equal loading of proteins.

### RNA isolation and quantitative RT-PCR

Total RNA isolation was performed using Trizol reagent (Beyotime, R0016) according to the manufacturer’s protocol. Reverse transcriptional PCR was done using PrimeScript RT reagent kit (TaKaRa, DRR037A). qPCR analysis was undertaken in Verti Thermal Cycler (Applied Biosystem) using SYBR Green Real Time PCR kit (TOYOBO, QPK-201). Data collection was carried out using a StepOne Plus Real-Time PCR System Thermal Cycling Block (Applied Biosystems). Primers for qPCR reactions were as follows:

- Bcl-2 (human): 5′-TTGAGGAAGTGAACATTTCGGTG-3′, 5′-AGGTTCTGCGGACTTCGGTC-3′;
- PUMA (human): 5′-GACCTCAACGCACAGTA-3′, 5′-CTAATTGGGCTCCATCT-3′;
- GAPDH (human): 5′-TGTTGCCATCAATGACCCCTT-3′, 5′-CTCCACGACGTACTCAGCG-3′.

### Calcium imaging

The calcium imaging was performed as previously described [27]. The cells were seeded in a 3.5 cm dish containing glass coverslips for 24 h and loaded with 10 μM Fluo-4-AM (Dojindo, Kumamoto, Japan) in PBS for 30 min. Then the cells were washed three times with PBS and observed under the microscope. 10 μM GF was added into the dish at 50 sec. The intracellular Ca^2+^ mobilization was monitored using a fluorescence microscope (IX83 system; Olympus, Tokyo, Japan) equipped with a band-path filter set (FITC; Olympus). The emission signal was recorded with a CCD camera. The fluorescent signals were recorded and analyzed using Olympus-analyzer software. The time courses of the fluorescence level of particular cells are expressed as the change in the fluorescent intensity normalized to the baseline-level fluorescence.

### Tumorigenesis in nude mice

Tumorigenesis in nude mice was previously described [28]. All animal studies were conducted according to protocols approved by the Shanghai University of Traditional Chinese Medicine Animal Care and Use committee (Certificate No. SYXK2-14-0008). Four-weeks-old male BALB/c nude mice were purchased from the Experimental Animal Center of Chinese Academy of Science (Shanghai, China). Approximately 1 × 10^6^ PC3 cells suspended in 100 μL of PBS and 100 μL of Matrigel (BD Biosciences) were injected s.c. into the right sides of the animals. One week later, 20 mice bearing tumors around 50 mm^3^ in volume were randomly divided into four groups (n = 5 per group): 1. Control (normally fed, receiving daily i.p. vehicle), 2. Caloric restriction (fed with 70% of their normal food intake, receiving daily i.p. vehicle), 3. GF (normally fed, receiving daily i.p. 20 mg/kg of GF), and 4. GF + caloric restriction (calorie-restricted mice receiving daily i.p. 10 mg/kg of GF). Mice were administered via intraperitoneal injection vehicle control solvent (0.5% DMSO, 0.5% Tween-80 in saline) and GF at the dose of 10 mg/kg or 20 mg/kg in 200 μl vehicle once every other day. Tumor size was monitored and measured by caliper measurements over a period of two weeks. The volume was calculated using the formula: 0.5 × width^2^ × length, width is the smallest side of the tumor. At the end of the experiment (16 days after treatment), the mice were sacrificed and their tumor weight was measured.

### Immunohistochemistry

Paraformadehyde-fixed, paraffin-embedded tumor specimens were processed with standard immunohistochemical (IHC) staining. The H&E staining was performed according to established protocols [25]. The tumor sections were treated in the following steps: hematoxylin for 10 min, 1% acid–ethanol for 30 s, 1% ammonia water for 30 s, and eosin for 10 s. After staining, the tissue section was dehydrated with water–ethanol–xylene gradients. IHC staining was performed according to a recently published protocol [10]. The primary antibodies were used as 1:200 for cleaved caspase-3 (Cell signaling, #9664).

### Statistical analysis

All data were given as mean ± standard deviation (SD) of three independent experiments. Student’s t-test was selected for the statistical analysis for comparison of the two groups. Values of *P* <0.05 were considered to be significant.

## Results

### Guttiferone F inhibits prostate cancer cell growth under nutrient deprivation

To identify effective compounds specifically targeting PCa, we performed a cell viability assay using LNCaP cells. We started screening from prenylated benzophenone, polycyclic polyprenylated acylphoroglucinols (PPAPs) and xanthones extracted from *Garcinia* species [29-31]. Among all the tested compounds, Guttiferone F (GF, Figure 1) exhibited the highest potency against LNCaP cells. Details on the extraction, isolation and identification of GF are in the Additional file 1. Notably, to investigate the effect of compounds under nutrient deprivation, the cell viability assay was performed in serum depleted medium [32]. We then examined the inhibitory effect of GF on several cancer cell lines. As shown in Table 1, GF displayed anti-proliferative effects against all the cancer cell lines. Interestingly, GF also showed the most potent activity against LNCaP cells with IC_50_ at 5.17 μM in serum-free medium, suggesting that GF sensitized PCa to nutrient starvation induced cell death. Therefore, we chose GF for further study to elucidate its mechanisms of action in inhibiting PCa growth during nutrient starvation.

**Table 1:** **IC**_**50**_ **values of GF on different cancer cell lines**

Cell line	Cell type	IC_50_(μM)
LNCaP	Prostate Cancer	5.17 ± 0.20
LNCaP	Prostate Cancer	20.00 ± 0.59 *
PC-3	Prostate Cancer	12.64 ± 3.01
Hep G2	Hepatocellular Carcinoma	32.93 ± 1.56
HeLa	Cervical Cancer	13.13 ± 1.32
CNE	Nasopharyngeal Carcinoma	17.97 ± 1.30

The IC_50_ values were determined in serum withdraw medium using MTT assay. GF inhibits the growth of different cancer cell lines selectively. 1× 10^4^ cells were plated in 96-well plates per well and treated with different dose of GF for 48 h. *The IC_50_s were obtained in cells under complete medium. Values are presented as mean ± s.e.m. of three independent experiments.

### Guttiferone F induces cell death in prostate cancer cells

**Figure 2:**
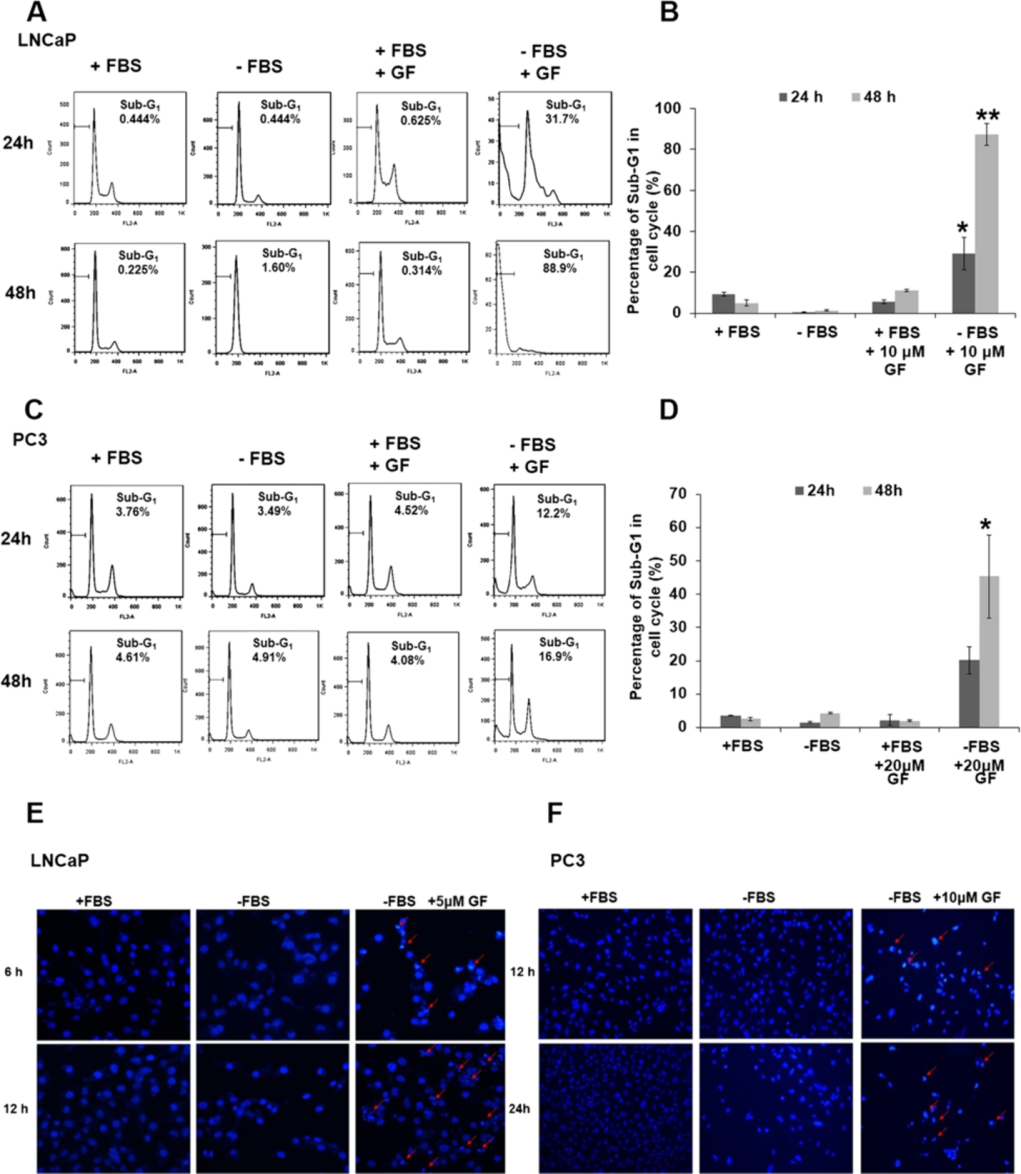
GF induces apoptosis in prostate cancer cells. (**A**) Flow cytometry analysis of sub-G1 population in GF-treated LNCaP cells. LNCaP cells were treated with GF (10 μM) cultured in either complete medium (RPMI1640 with serum) or nutrient-deprived medium (RPMI1640 without serum) for 24 h or 48 h. The cells were fixed and stained with propidium iodide (PI). (**B**) Statistic analysis of Sub-G1 fraction of samples in (**A**). (**C**) Flow cytometry analysis of sub-G1 population in GF-treated PC3 cells. PC3 cells were treated with GF (20 μM) cultured in either complete medium (RPMI1640 with serum) or nutrient-deprived medium (RPMI1640 without serum) for 24 h or 48 h. (**D**) Statistic analysis of Sub-G1 fraction of samples in (**C**). Data are presented as means ± SD from three independent experiments and analyzed using Student’s t-test (*P <0.05, **P <0.01). (**E** and **F**) Nuclear morphology changes in GF treated LNCaP cells (**E**) or PC3 cells (**F**). Cells were treated with GF (5 μM for LNCaP, 10 μM for PC3) cultured in either complete medium (RPMI1640 with serum) or nutrient-deprived medium (RPMI1640 without serum) for 6 h or 12 h. Arrows pointed to typical chromatin condensation and DNA fragmentation.

**Figure 3:**
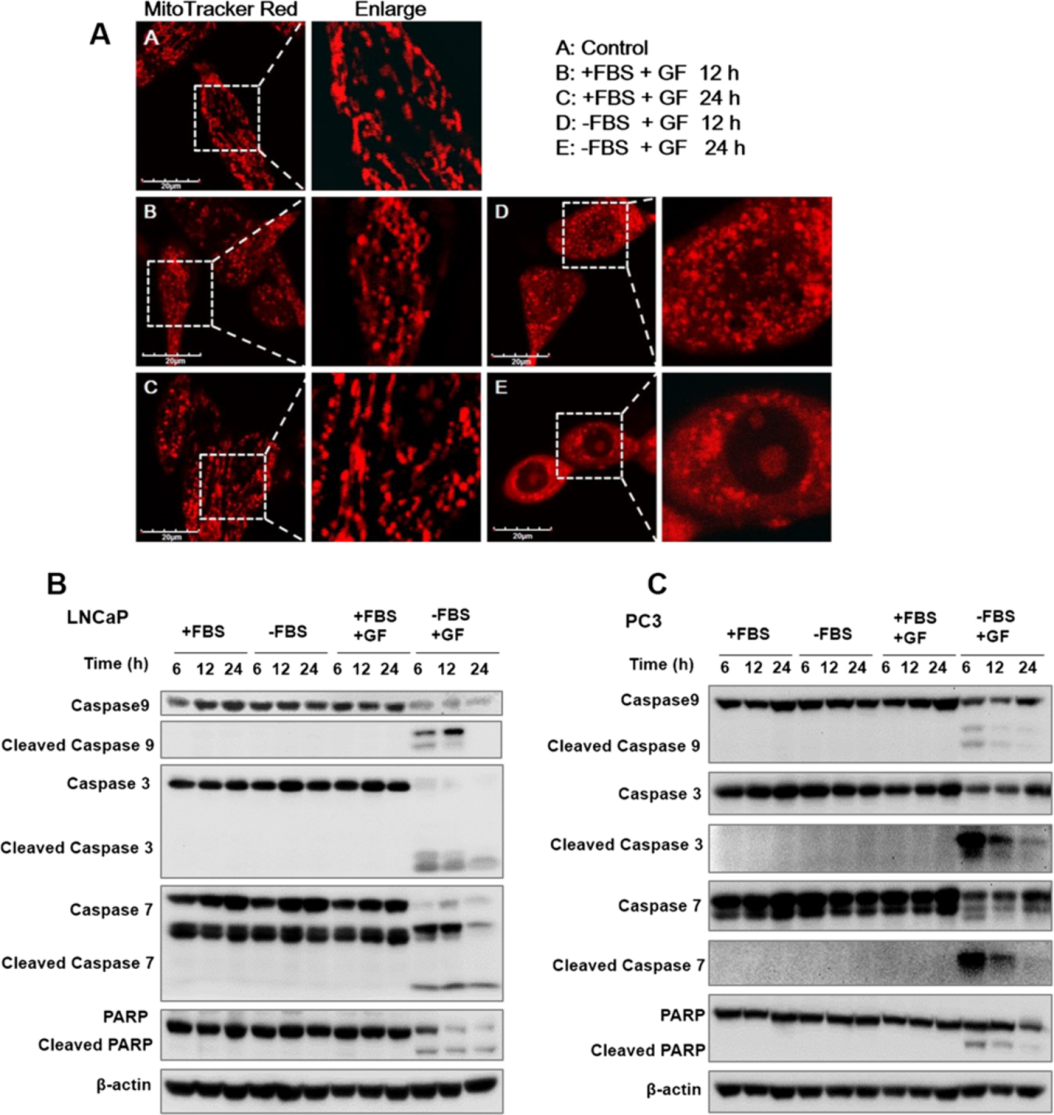
GF triggers mitochondria dysfunction and caspases activation. (**A**) Mito-Tracker Red staining shows the mitochondrial morphology and potential changes upon GF treatment. The LNCaP cells were treated with 10 μM GF for 12 h and 24 h. After GF treatment in serum-free medium, changes in mitochondria were scanned by laser confocal microscopy. Scale bar, 20 μm. (**B**) Western blot showed that the activation of caspase 3, 7, 9 and PARP protein in LNCaP cells treated with 10 μM for different time. GF triggers mitochondia-mediated apoptosis in LNCaP cells. (**C**) Western blot showed that the activation of caspase 3, 7, 9 and PARP protein in PC3 cells treated with 10 μM GF for different time.

To investigate the mechanism by which GF affect PCa in nutrient starvation, we performed flow cytometry to quantify the sub-G1 population. Treatment with GF alone (10 μM) in complete medium (RPMI1640) did not show potency to LNCaP cells. Whilst nearly 31% and 88% sub-G1 cells were detected at 24 h and 48 h in serum-free medium, respectively (Figure 2A and B). In addition, PC3 cells treated with GF (20 μM) for 24 h and 48 h in serum-free medium showed an increase in sub-G_1_ fraction (Figure 2C and D). To better characterize the effect of GF on DNA content, we examined the DNA morphology changes using 4,6-diamidino-2-phenylindole (DAPI) staining. As shown in Figure 3E and F, the cells treated with GF in serum-free medium displayed chromatin condensation and DNA fragmentation, suggesting the presence of apoptotic cells. The control LNCaP and PC3 cells maintained homogeneous chromatin distribution. Taken together, our results suggested that GF induced PCa cell death in serum-free medium, resulting in chromatin condensation and DNA fragmentation.

### Guttiferone F triggers mitochondria-mediated and caspase-dependent apoptosis

Previously, we showed that GF could induce apoptosis using our caspase-sensor based high-throughput assay in HeLa cells [24]. Here, we performed more detailed apoptotic analysis on GF in PCa. The key events of mitochondrial pathway including mitochondrial fission, swelling, and reduction of mitochondrial membrane potential (Δψ_m_) were examined in GF-induced apoptosis. We stained cells with a fluorescent dye, MitoTracker Red, to observe the mitochondrial morphology during GF treatment. The distribution and intensity of MitoTracker Red can thus be used to reveal mitochondrial morphology and indicate the integrity of mitochondrial structure [25]. Control LNCaP cells displayed mitochondria clustered around the nucleus. However, as shown in Figure 3A, LNCaP cells treated with 10 μM GF in serum depleted medium displayed mitochondria fission and swelling. To investigate the effect of GF on mitochondrial membrane potential (Δψ_m_), we stained PC3 cells in the absence or the presence of GF in serum-free medium with TMRE and detect the fluorescent intensity using flow cytometry. As shown in Additional file 2: Figure S1, GF attenuated the mitochondrial membrane potential in serum-free medium in a short period treatment. Furthermore, we analyzed the extent of caspases and poly-ADP ribose polymerase (PARP) cleavage in LNCaP cells after 10 μM GF treatment by Western blotting. Our results showed the activation of caspase-9, −7 and −3 and the production of cleaved PARP at 6, 12 and 24 h of GF treatment in serum depleted medium. Interestingly, the starvation or GF treatment alone did not show apoptotic activation, suggesting that GF could sensitize PCa cells to nutrient deprivation induced cell death. In PC3 cells treated with GF and serum depletion, the activation of caspases family and cleavage of PARP were also detected (Figure 3C).

**Figure 4:**
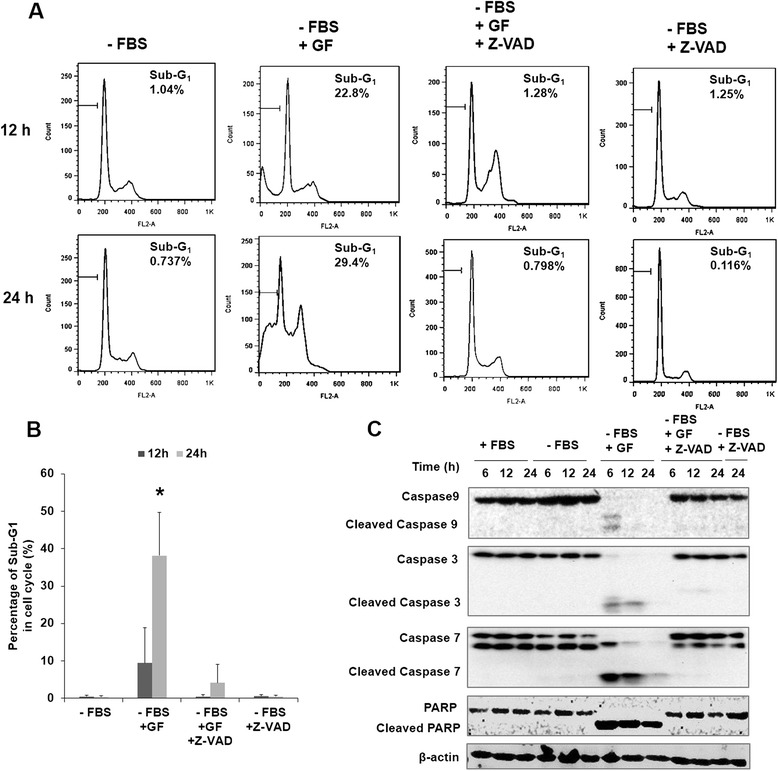
GF induces apoptosis in serum-free medium. (**A** and **B**) Effects of 10 μM GF or 20 mM Z-VAD-fmk on cell cycle in LNCaP cells after 24 h and 48 h treatment. Cytometric analyses were performed using PI staining. (**C**) Western blot showed that the activation of caspase 3, 7, 9 and PARP protein in LNCaP cells treated with 10 μM GF or 20 mM Z-VAD-fmk for different time. *Significant (*P* <0.05); **highly significant (*P* <0.001).

To further investigate the role of caspase activation in GF-induced apoptosis, the effect of pan-caspase inhibitor Z-VAD-fmk in preventing GF-induced cell death was examined. As shown in Figure 4A and B, when LNCaP cells were treated with 10 μM GF without serum, the percentage of cell death reached 22.8% and 19.4% at 12 h and 24 h, respectively. However, when cells were pre-treated with caspase inhibitor Z-VAD-fmk for 1 h, the percentage of cells death reduced to less than 2% after 12 and 24 h. Moreover, western blots showed that cleavage of caspase 3, 7, 9 and PARP were also inhibited (Figure 4C).

### Guttiferone F regulates Bcl-2 family proteins, MAPK pathway, and androgen receptor

**Figure 5:**
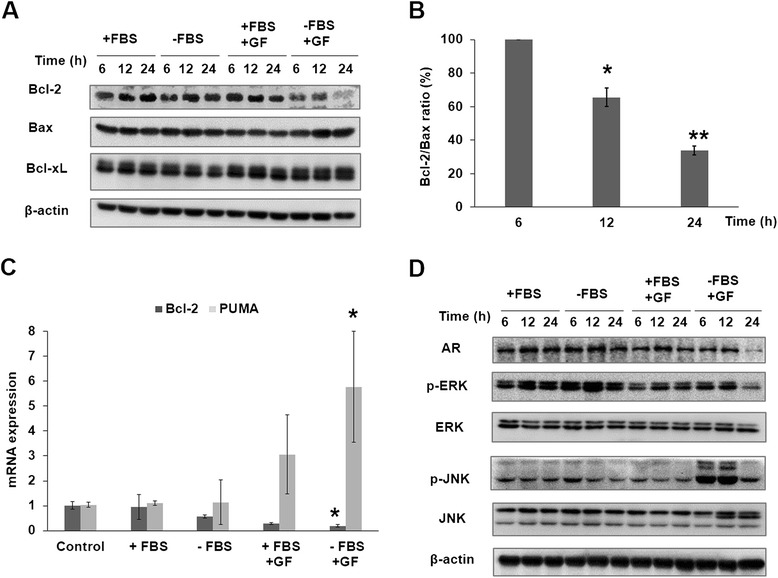
GF affected Bcl-2 family, MAPKs, and androgen receptor (AR). LNCaP cells were treated with 10 μM GF for 6, 12 and 24 h, respectively. (**A**) Western blot showed the expression of Bcl-2, Bax and Bcl-xL in LNCaP cells treated with 10 μM GF with or without FBS. (**B**) The ratio of Bcl-2/Bax protein expression. (**C**) Relative Bcl-2 and PUMA mRNA level (compared with GAPDH) was analyzed by quantitative RT-PCR. LNCaP cells were treated for 0 h as control. Cells were treated with GF (10 μM) in serum-free medium or serum medium for 6 h. * indicates a significant difference from the controls. *p* < 0.05. (**D**) The protein expression of MAPKs and AR, phosphorylated ERK and phosphorylated ERK in LNCaP cells treated with 10 μM GF with or without FBS.

Bcl-2 family proteins, such as Bcl-2 and Bax, are upstream signals of caspases activation and play important roles in regulating mitochondria related apoptosis [33]. We then explored the effect of GF on Bax, Bcl-2 and Bcl-xL to determine the effects of GF on mitochondria dysfunction. As illustrated in Figure 5A and B, LNCaP cells treated with GF in serum depleted medium had a significantly lower level of Bcl-2 and higher level of Bax than those in complete medium, whilst the level of Bcl-xL was not significantly different. Taken together, these results suggested that GF might induce apoptosis of LNCaP cells through Bcl-2 degradation. To determine whether regulation of the Bcl-2 protein was mediated via modulating expression of mRNA, we analyzed the expression of the transcription coding by RT-PCR procedures. Figure 5C shows that GF express mRNA for the Bcl-2 as mediator of apoptosis, in agreement with their protein levels. GF also improved mRNA for PUMA which is a pro-apoptotic gene.

The mitogen-activated protein kinase (MAPK) pathways are involved in the regulation of diverse cellular events, including proliferation, differentiation and apoptosis. These pathways involve c-Jun-N-terminal kinase (JNK), extracellular signal-regulated protein kinase (ERK). Normally, the ERK module plays a cytoprotective role, whereas the JNK exerts pro-apoptotic functions [34,35]. Therefore, we speculate that MAPK pathway may play an important role in GF-induced apoptosis. We then examined the expressions of ERK and JNK proteins and phosphorylation level under GF treatment (Figure 5D). Our results showed that GF could up-regulate the p-JNK expressions and down-regulate the p-ERK expressions. Thus, JNK and ERK may play critical roles in the GF-induced apoptosis.

Androgen receptor (AR) is the key indicator for prostate cancer prognosis [3]. We also analyzed the expression of AR in LNCaP cells treated with GF. As shown in Figure 5D, LNCaP cells treated with GF in serum depleted medium resulted in a significant reduction in AR expression.

### Guttiferone F induces cytosolic Ca2+ elevation in prostate cancer cells

**Figure 6:**
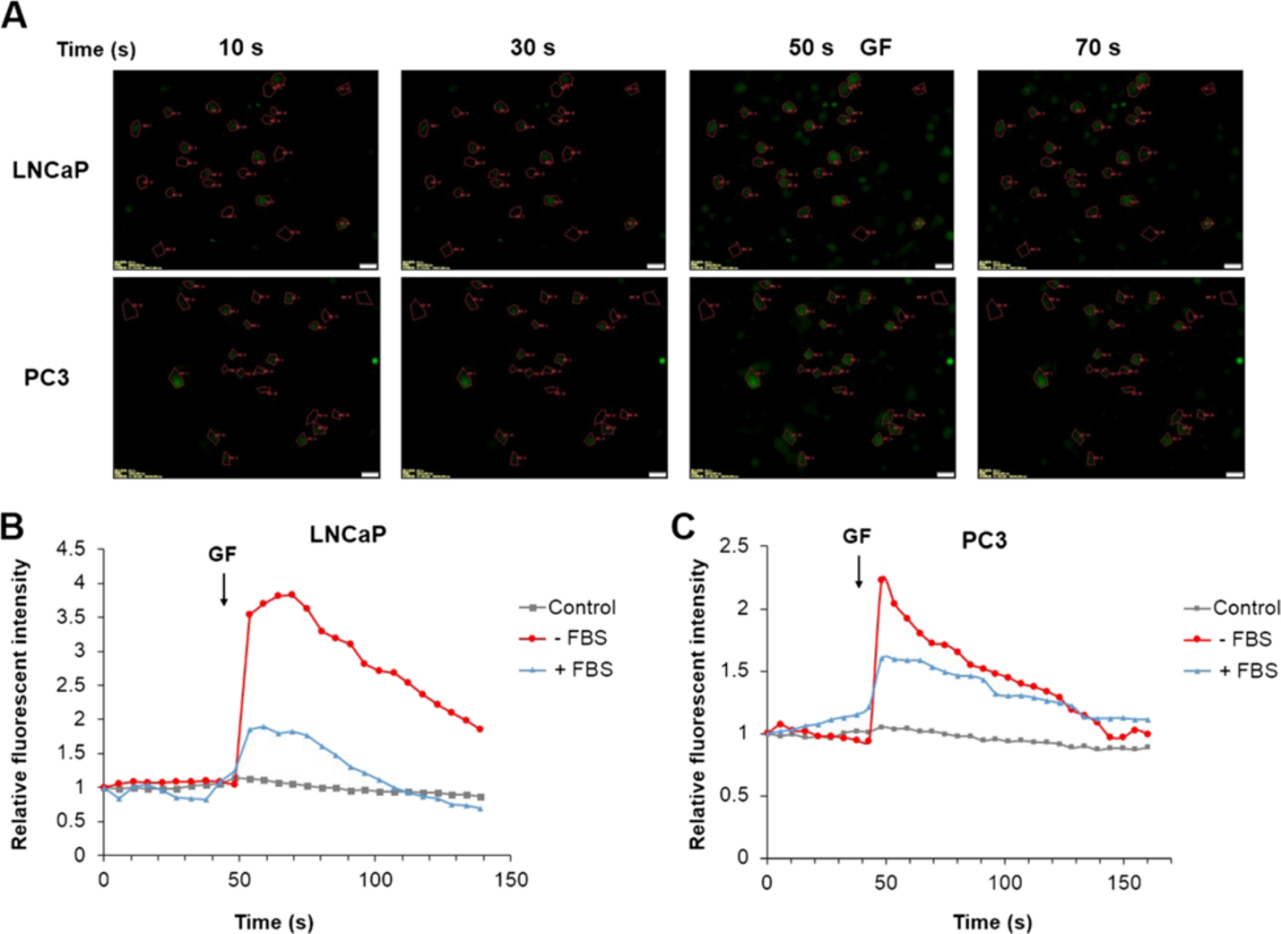
Effects of GF on cytosolic Ca^2+^ signal. (**A**) Fluorescent Images for LNCaP and PC3 cells treated with GF. ROI 1–20 were selected cells; ROI 21–23 were selected as background. Fluorescent intensity was quantified by background subtracted and normalized from the start. Cells were stained with Fluo-4-AM, and images were acquired by live cell imaging system (Ex: 488 nm, Em: 543 nm BP), for 3 min with 5 sec interval. GF was added at 50 sec and the arrows showed the addition of 10 μM GF. (**B**) Time-response curve (mean) was determined from data generated in selected 20 LNCaP cells; (**C**) Time-response curve (mean) was determined from data generated in selected 20 PC3 cells. Experiments were repeated 3 times.

Calcium ion (Ca^2+^), an important cellular regulator, has an important role in the fate of the cell. Deregulation of calcium homeostasis is toxic to cells and may induce cell death [36-38]. In addition, ER stress, which is partially induced by calcium depletion, could modulate MAPK signaling pathways and cell fates, including cell cycle, cell survival or cell death [39]. We then investigated the effect of GF on cytosolic Ca^2+^ concentration using fluorescent Ca^2+^ indicator, Fluo-4-AM. As shown in Figure 6A, GF could trigger strong Ca^2+^ elevation in LNCaP and PC3 cells. The fluorescent intensity dramatically increased soon after the addition of GF into the culture medium. To quantify the effect of GF on Ca^2+^ signal, we calculated the change in relative Ca^2+^ intensity in multiple cells. Interestingly, we detected stronger Ca^2+^ peak in the serum-free medium in both LNCaP and PC3 cells (Figure 6B and C). Our results suggested that prostate cancer cells showed stronger response on Ca^2+^ signal in serum-free medium upon GF treatment, which might be responsible for the activation of the MAPK pathways.

To further evaluate the functional roles of Ca^2+^ elevation and JNK activation in GF induced cell death, we applied Ca^2+^ chelator BAPTA-AM and JNK inhibitor SP600125 on PC3 cells upon GF treatment. As shown in Additional file 2: Figure S2, both BAPTA-AM and SP600125 attenuated the GF-induced sub-G1 distribution, suggesting that Ca^2+^ and JNK signals played important roles on GF mediated cell death.

### Caloric restriction enhances the antitumor activity of Guttiferone F *in vivo*

**Figure 7:**
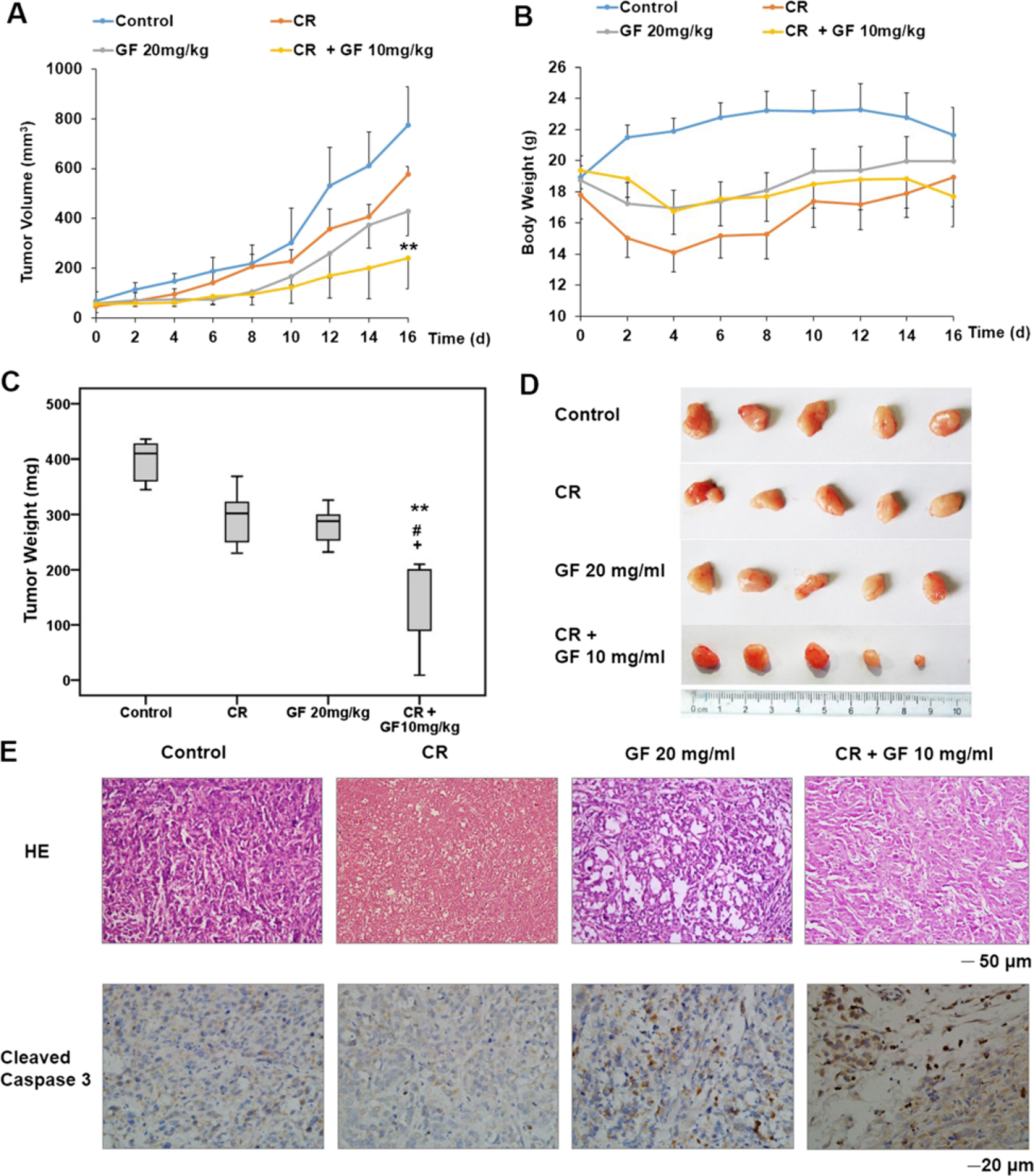
GF exhibits anticancer activity in PCa xenograft. (**A**) Four weeks old nude mice were engrafted with PC3 cells and observed until tumors reached ~50 mm^3^. Tumor-bearing mice were then treated vehicle, GF (20 mg/kg) by intraperitoneal once every 2 d for a total of eight injections. (**B**) Body weight of the mice. (**C**) Mice were sacrificed and tumors were resected and weighed 2 d after the final injection. Control (mean: 395.8 mg; median: 410.0 mg; range: 345.0–436.0 mg), CR (mean: 296.0 mg; median: 302.0 mg; range: 230.0–369.0 mg), GF (mean: 279.8 mg; median: 288.0 mg; range: 232.0–326.0 mg) and CR + GF (mean: 141.8 mg; median: 200.0 mg; range: 9.0–210.0 mg). Error bars represents maximum and minimum; boxes represents the upper and lower quartiles and median. ** *P <*0.01 comparing with Control; ^**#**^*P* <0.05 comparing with CR; ^**+**^*P* <0.05 comparing with GF 20 mg/kg. The statistic analysis was performed by Student’s t-test. (**D**) Images for representative tumor from (**C**). (**E**) Histological stains (H&E staining) of tumor cross-sections from mice treated with control, CR, GF and GF + CR. Images were taken at 200X magnification. Scale bar, 50 μm. Immunohistochemical staining for cleaved Caspase3 in tumor sections. Images were taken at 400X magnification. Scale bar, 20 μm.

To determine the antitumor activity of GF *in vivo*, nude mice were injected with PC3 and then administrated GF, or vehicle control. Tumor volume and mass increased dramatically in the control group, whereas tumor growth was significantly less prominent in GF-treated mice (Figure 7A, C and D). GF inhibited PCa cell proliferation and induced apoptosis in serum-free medium. Therefore, we investigated the therapeutic potential of combined treatment with GF and caloric restriction (CR) in xenograft mouse model. The xenograft tumor was passed through subcutaneous injection of PC3 cells suspension. When the tumor size reached ~50 mm^3^, mice were divided into control, CR, GF (20 mg/kg) and GF (10 mg/kg) and CR groups. All CR mice received daily meals amounting to 70% of the caloric intake of their *ad libitum* counterparts [40]. Interestingly, in animals exposed to a combination of GF and CR, tumor size and tumor weight were lower than other groups (Figure 7A, C and D).

H&E staining showed that the cells were densely packed in the tumor tissue of the control mice. The cell density was significantly reduced with the formation of many vacuoles in the tumor tissue of GF-treated or GF and CR treated mice (Figure 6E). Finally, we performed IHC to detect Caspase3 activation in tumor tissues. As shown in Figure 6E, an increase of cleaved Caspase3 staining was observed in tumor tissue from both GF alone and GF and CR groups, indicating that CR enhance GF-induced Caspase3 dependent apoptosis *in vivo.*

## Discussion

Oxygen and nutrient supply to tissues are pivotal to cancer cell survival and development. Compared to normal cells, tumor cells proliferate more rapidly due to the unregulated cell cycle caused by genome mutation. Many of the tumor cells are in a state of nutrient deprivation before angiogenesis but have the ability to survive under extreme conditions such as low nutrient and oxygen supply [41]. Therefore, novel effective compounds targeting cancer cells under nutrient deprivation are essential candidates for anticancer drugs [32]. In this study, we used serum free medium to mimic the low nutrient environment to screen active compounds from the *Garcinia* compounds library. We found that GF showed significant inhibitory effect against PCa, including androgen-dependent PCa cells LNCaP and androgen-independent PCa cells PC3. We then investigated the working mechanism of GF using multiple approaches. Our data suggested that GF could enhance the cell’s influx of internal calcium and induce caspases dependent apoptosis in both LNCaP and PC3 cells under serum depleted but not complete medium. Serum starvation is a general model for investigating the molecular mechanisms underlying apoptosis and autophagy, which generally function as defense strategies when cells are under oxygen and nutrient stress [42,43]. Consistent with our cell viability assay, the apoptotic events including sub-G1 fraction increase, caspases activation, PARP cleavage and ratio of Bcl-2/Bax decrease were observed in GF treated starved PCa. The cells cultured in complete medium did not respond to GF treatment, which implied that GF might be not toxic to cells under normal nutrient environment. The induction of autophagy plays a protective role in helping the cells escape from stresses such as hypoxia, nutrient deprivation and chemotherapy. It would be interesting to investigate the detailed mechanisms in which GF can sensitize the PCa cells to nutrient deprivation induced cell death. It is also notable that GF exhibits variant activities to different cancer cells, including prostate cancer cells LNCaP and PC3 cells. PC3 cell line is p53 deficient and LNCaP contains p53, which can cause different response to chemotherapeutic molecules [44]. In our earlier study, GF showed anti-cancer activity against another p53 deficient cell line HeLa [24]. These results suggested that the mechanism of action of GF might relate to p53 signaling pathway. It will be also interested to investigate the synergetic effect of GF with some p53 activator such as DNA damage agents.

The polycyclic polyprenylated acylphloroglucinols (PPAPs), a class of secondary metabolites derived from plants of the *Guttiferae* family, have drawn researcher’s attention because of their diverse biological activities. Nemorosone, extracted from flowers of the *Clusia* species, was found to activate the unfolded protein response (UPR) and elevate cytosolic Ca^2+^ in MIA-PaCa-2 pancreatic cancer cells [45]. In addition, it was reported that the toxicity of nemorosone on estrogen receptor positive breast cancer cell (MCF7) was partially due to its effect on phosphorylation of ERK and AKT but not related to calcium signaling [46]. In starved 3T3 fibroblasts, addition of serum could induce cytosolic calcium flux, followed with the increase in p42/p44 MAPK phosphorylation, which activated cell cycle progression [47]. In our study, GF reduced androgen receptor expression, and regulated JNK and ERK phosphorylation in LNCaP cells under serum starvation. Furthermore, the addition of GF to culture medium caused a significant Ca^2+^ elevation, which was higher in serum depleted medium than in full medium. It is still unclear whether the Ca^2+^ signal contributes to the activation of MAPK pathway and cell death. The mechanism of GF activation of Ca^2+^ should be further investigated in the future.

During carcinogenesis, adaptation of the cancer cells to their tumor microenvironments is pivotal to tumor growth and progression. Therefore, it is reasonable to consider agents that inhibit this adaptation of the cells to their microenvironment as potential antitumor agents. In our animal study, GF suppressed PC3 engrafted tumor growth. The level of inhibition was significantly enhanced when the animals were under caloric restriction diet. In addition, we found that 20 mg/kg GF exhibited high potency to mice under caloric restriction (data not shown), suggesting that GF and caloric restriction had strong interaction and synergetic effects in animal model. All together, these interesting findings suggest that controlling the diet during chemotherapy may have additional beneficial effects. It is still a question if the serum free environment *in vitro* can mimic the caloric restriction *in vivo*. As far as we know, serum contains many nutrients including growth factors, proteins, lipids etc. Lack of these factors causes nutrient stress to cultured cells and activates many signaling pathways including autophagy. Caloric restriction *in vivo* is far more complicated than the serum withdraw and results in many phenotypes, such as autophagy induction and acetyle transferases inhibition [48]. Nevertheless, autophagy induction is one of the important signals mediating these two models. Therefore, further study is necessary to elucidate the correlation between prostate cancer, autophagy, and dietary habits.

## Conclusions

In summary, we screened toxic compound against prostate cancer cells in serum withdraw medium. We found that the natural compound Guttiferone F induced prostate cancer cell apoptosis under serum starvation *via* calcium and JNK elevation. Combined with caloric restriction, Guttiferone F exerted significant growth inhibition of xenograft model using PC3 cells*.* These findings suggest that Guttiferone F is therefore a potential anti-cancer compound and caloric restriction can enhance the efficacy of Guttiferone F *in vivo*.

## Additional files

Additional file 1:
**Supplementary information.**

Additional file 2: Figure S1. Guttiferone F decreases mitochondrial membrane potential under serum deprivation. PC3 cells were treated with or without GF in the presence or the absence of serum for 6 h. Cells then were collected and stained by 50 nM TMRE for 10 min and analyzed by flow cytometry. Red line shows that GF reduces TMRE staining under serum withdraw condition, indicating that GF decreases mitochondrial membrane potential. **Figure S2.** Calcium chelator BAPTA-AM and JNK inhibitor SP600125 attenuate GF-induced cell death in serum deprivation. PC3 cells were pre-incubated in 10 μM BAPTA-AM or 20 μM JNK inhibitor for 1 h, then treated in the absence (upper panel) or the presence (lower panel) of GF for 24 h. The cells were collected and analyzed by PI staining for sub-G1 distributions. All samples were cultured in serum free medium in this experiment.

## Abbreviations

AR: Androgen receptor
CR: Caloric restriction
FBS: Fetal bovine serum
GF: Guttiferone F
JNK: c-Jun N-terminal kinases
PCa: Prostate cancer
PARP: Poly ADP ribose polymerase
