# Supplementary material for: The natural compound Guttiferone F sensitizes prostate cancer to starvation induced apoptosis via calcium and JNK elevation
Source: BMC Cancer. 2015 Apr 11;15:254. doi: 10.1186/s12885-015-1292-z (PMC4394563; doi:10.1186/s12885-015-1292-z)
Supplement: Additional file 1: — Supplementary information. [file 12885_2015_1292_MOESM1_ESM.pdf]

Supplementary Information

Plant Material

Garcinia esculenta Y. H. Li twigs were collected in August 2010 in Nujiang, Yunnan Province, People's Republic of China. Plant material was identified by Prof. Yuanchuan Zhou, Yunnan University of Traditional Chinese Medicine. A voucher specimen (Herbarium No. 20100801) has been deposited at the Innovative Research Laboratory of TCM, Shanghai University of Traditional Chinese Medicine.

Extraction, Isolation and Identification of Guttiferone F

Air-dried and powdered twigs of the plant (4 kg) were extracted with petroleum ether (5 × 20 L, two days each). The combined extracts were evaporated to dryness under vacuum to give fraction I (40 g). The remaining materials were refluxed with 80% EtOH (v/v, 5 × 20 L). The combined extracts were evaporated to dryness under vacuum, and the residue was suspended in H_2O (5 L) and extracted with EtOAc (5 × 5 L) to give fractions II (50 g, EtOAc soluble) and III (the aqueous fraction), respectively. The remaining materials were refluxed with distilled water (5 × 20 L) to give the water-soluble fraction (IV). Fraction I (37 g) was chromatographed on a silica gel column (CC) using a gradient of petroleum ether–EtOAc (100:0 to 50:50, v/v) guided by TLC, yielding fifteen fractions (IA–IO). Fraction IL was chromatographed on MCI gel eluted successively with 90% and 100% EtOH to afford two subfractions (IL1 and IL2). Fraction IL1 (10.5 g) was subjected to reverse-phase C_{18} silica gel CC, eluted in a step gradient manner with MeOH-H_2O (70:30 to 100:0) to yield Guttiferone F (350 mg). The structure of Guttiferone F was elucidated as

shown (Figure 1) by comparison of their spectroscopic data with published data.[23] The purity of Guttiferone F was checked by UPLC-DAD and the result displayed purity of above 98%.

Supplementary Chemicals

Calcium chelator BAPTA-AM (Cat. A1076) and JNK inhibitor SP600125 (Cat. S5567) were purchased from Sigma-Aldrich. TMRE, (Tetramethylrhodamine, Ethyl Ester, Perchlorate, Cat. T-669) was purchased from Molecular Probes.
