# Supplementary material for: The natural compound Guttiferone F sensitizes prostate cancer to starvation induced apoptosis via calcium and JNK elevation
Source: BMC Cancer. 2015 Apr 11;15:254. doi: 10.1186/s12885-015-1292-z (PMC4394563; doi:10.1186/s12885-015-1292-z)
Supplement: Additional file 2: Figure S1. — Guttiferone F decreases mitochondrial membrane potential under serum deprivation. PC3 cells were treated with or without GF in the presence or the absence of serum for 6 h. Cells then were collected and stained by 50 nM TMRE for 10 min and analyzed by flow cytometry. Red line shows that GF reduces TMRE staining under serum withdraw condition, indicating that GF decreases mitochondrial membrane potential. Figure S2. Calcium chelator BAPTA-AM and JNK inhibitor SP600125 attenuate GF-induced cell death in serum deprivation. PC3 cells were pre-incubated in 10 μM BAPTA-AM or 20 μM JNK inhibitor for 1 h, then treated in the absence (upper panel) or the presence (lower panel) of GF for 24 h. The cells were collected and analyzed by PI staining for sub-G1 distributions. All samples were cultured in serum free medium in this experiment. [file 12885_2015_1292_MOESM2_ESM.pdf]

Supplementary Figure 1.

Supplementary Figure 1

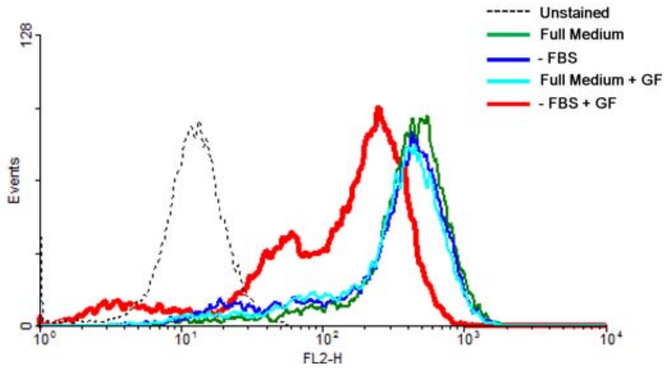

Supplementary Figure 1. Guttiferone F decreases mitochondrial membrane potential under serum deprivation. PC3 cells were treated with or without GF in the presence or the absence of serum for 6 h. Cells then were collected and stained by 50 nM TMRE for 10 min and analyzed by flow cytometry. Red line shows that GF reduces TMRE staining under serum withdraw condition, indicating that GF decreases mitochondrial membrane potential.

Supplementary Figure 2.

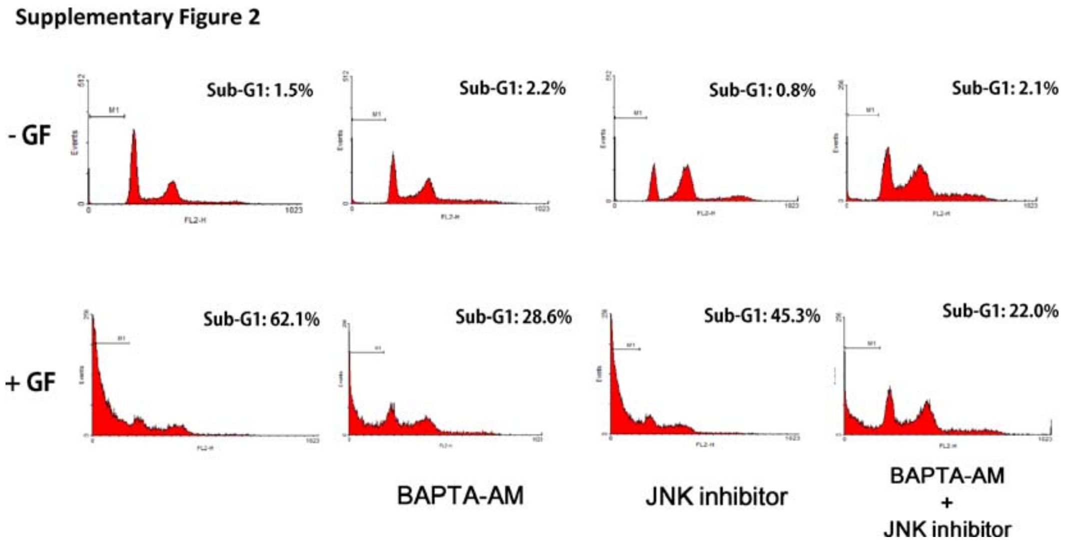

Supplementary Figure 2. Calcium chelator BAPTA-AM and JNK inhibitor SP600125 attenuate GF-induced cell death in serum deprivation. PC3 cells were pre-incubated in 10 µM BAPTA-AM or 20 µM JNK inhibitor for 1 h, then treated in the absence (upper panel) or the presence (lower panel) of GF for 24 h. The cells were collected and analyzed by PI staining for sub-G1 distributions. All samples were cultured in serum free medium in this experiment.
